# Aortic Remodeling Kinetics in Response to Coarctation-Induced Mechanical Perturbations

**DOI:** 10.3390/biomedicines11071817

**Published:** 2023-06-25

**Authors:** Arash Ghorbannia, Mehdi Maadooliat, Ronald K. Woods, Said H. Audi, Brandon J. Tefft, Claudio Chiastra, El Sayed H. Ibrahim, John F. LaDisa

**Affiliations:** 1 Joint Department of Biomedical Engineering, Medical College of Wisconsin, Marquette University, Milwaukee, WI 53226, USA; said.audi@marquette.edu (S.H.A.); btefft@mcw.edu (B.J.T.); eibrahim@mcw.edu (E.S.H.I.); jladisa@mcw.edu (J.F.L.); 2 Section of Pediatric Cardiology, Department of Pediatrics, Medical College of Wisconsin, Milwaukee, WI 53226, USA; 3 Herma Heart Institute, Children’s Wisconsin, Milwaukee, WI 53226, USA; 4 Department of Mathematical and Statistical Sciences, Marquette University, Milwaukee, WI 53233, USA; mehdi.maadooliat@marquette.edu; 5 Division of Pediatric Cardiothoracic Surgery, Department of Surgery, Medical College of Wisconsin, Herma Heart Institute, Children’s Wisconsin, Milwaukee, WI 53226, USA; rwoods@mcw.edu; 6 PoliTo^BIO^Med Lab, Department of Mechanical and Aerospace Engineering, Politecnico di Torino, 10129 Turin, Italy; claudio.chiastra@polito.it; 7 Department of Radiology, Medical College of Wisconsin, Milwaukee, WI 53226, USA; 8 Department of Physiology, Medical College of Wisconsin, Milwaukee, WI 53226, USA

**Keywords:** pathological remodeling, arterial adaptation, homeostasis, hypertension, smooth muscle cell proliferation, coarctation of the aorta

## Abstract

**Background:** Coarctation of the aorta (CoA; constriction of the proximal descending thoracic aorta) is among the most common congenital cardiovascular defects. Coarctation-induced mechanical perturbations trigger a cycle of mechano-transduction events leading to irreversible precursors of hypertension including arterial thickening, stiffening, and vasoactive dysfunction in proximal conduit arteries. This study sought to identify kinetics of the stress-mediated compensatory response leading to these alterations using a preclinical rabbit model of CoA. **Methods:** A prior growth and remodeling (G&R) framework was reformulated and fit to empirical measurements from CoA rabbits classified into one control and nine CoA groups of various severities and durations (n = 63, 5–11/group). Empirical measurements included Doppler ultrasound imaging, uniaxial extension testing, catheter-based blood pressure, and wire myography, yielding the time evolution of arterial thickening, stiffening, and vasoactive dysfunction required to fit G&R constitutive parameters. **Results:** Excellent agreement was observed between model predictions and observed patterns of arterial thickening, stiffening, and dysfunction among all CoA groups. For example, predicted vascular impairment was not significantly different from empirical observations via wire myography (*p*-value > 0.13). Specifically, 48% and 45% impairment was observed in smooth muscle contraction and endothelial-dependent relaxation, respectively, which were accurately predicted using the G&R model. **Conclusions:** The resulting G&R model, for the first time, allows for prediction of hypertension precursors at neonatal ages that is currently challenging to examine in preclinical models. These findings provide a validated computational tool for prediction of persistent arterial dysfunction and identification of revised severity–duration thresholds that may ultimately avoid hypertension from CoA.

## 1. Introduction

Coarctation of the aorta (CoA) is a constriction of the proximal descending thoracic aorta and is one of the most common congenital cardiovascular defects [1]. Surgical repair remains the standard approach for treatment in most young infants, while catheter intervention with balloon angioplasty and/or stenting is the standard of care in older children and adults [2,3]. Guidelines for intervention include a transcatheter peak-to-peak blood pressure (BP) gradient ≥20 mmHg, and many published reports regard this criterion as a hemodynamically significant CoA [3]. Unfortunately, hypertension and left-ventricular hypertrophy are common despite successful CoA repair based upon this threshold [4,5].

Mechanistic understanding of hypertension in CoA is hindered by the paucity of data to investigate changes in the kinetics of growth and remodeling (G&R) associated with coarctation-induced mechanical stimuli [6,7]. Hence, animal models are used to investigate underling mechanisms and precursors of hypertension in response to CoA [8]. In contrast to the current putative threshold, recent studies using a rabbit model of CoA suggest that precursors of hypertension are present among treated rabbits with CoA gradients <20 mmHg [8,9,10]. This finding raises doubts about the efficacy of the current guidelines in preventing refractory hypertension after treatment. Interestingly, the duration of coarctation-induced mechanical stimuli also correlates with hypertension precursors [7,9], further supporting the importance of earlier treatment that has been shown to reduce hypertension prevalence among treated CoA patients [11].

In general, the arterial wall is assumed to adapt to mechanical stimuli (perturbation from normal state) and recover a homeostatic state via smooth muscle (SM) cell synthesis and matrix turnover [12,13,14]. For example, in CoA, the presence of the narrowing can cause elevated BP proximally and a turbulent velocity jet distally. This perturbation in blood pressure-induced intramural wall stress (IWS: σ) proximally and blood flow-induced wall shear stress (WSS: ϕ) distally changes the vascular tone toward recovering IWS and WSS back to preferred ranges that are assumed to be ‘known’ to the local vasculature. Consequently, fibrillar proteins such as collagen and SM are deposited to remodel the extracellular matrix and modify thickness, arterial caliber, and material properties. The kinematics of this compensatory thickening/stiffening response is stress-driven and has been previously quantified as a linear function of deviations from homeostatic values [14,15,16].

The aim of this work is to quantify G&R parameters applicable to CoA using a computational approach tuned to replicate arterial thickening, stiffening, and dysfunction measured in rabbit aortas exposed to mechanical stimuli within ranges seen clinically. Completion of the study objective will provide a validated computational tool for prediction of persistent arterial dysfunction and allow for the much-needed identification of severity–duration thresholds that prevent arterial dysfunctions contributing to refractory hypertension among treated CoA patients.

## 2. Material and Methods

### 2.1. Stress-Mediated G&R Formulation

For a section of the aorta, at any time t, the current configuration can be represented simply as a pressurized cylindrical thin-wall tube. Deformation can, therefore, be described by mapping the reference point (R, Θ, Z) onto a new position (r,θ,z) in the same coordinate system when deformed:

(1) $$r=r\left(R\right),\;\;\theta =\Theta ,\;\;z=\kappa Z,$$

for some function $r\left(R\right)$ and parameter κ. That is, deformations are restricted so that the principal axes of deformation coincide with the coordinate directions of the principal stretches.

(2) $$\lambda_{r}=\frac{\partial r}{\partial R},\;\;\lambda_{\theta }=\frac{r}{R},\;\;\lambda_{z}=\kappa .$$

Assuming incompressibility, i.e., $\lambda_{\theta }\lambda_{z}\lambda_{r}=1$, one can reduce the number of independent stretch parameters to $\lambda_{\theta }=\lambda$ and $\lambda_{z}$

(3) $$\lambda_{r}={\left(\lambda \lambda_{z}\right)}^{-1}=\frac{1}{\kappa \lambda }.$$

Equilibrium in the radial direction gives the following [17]:

(4) $$\frac{\partial \sigma_{r}\left(t\right)}{\partial r}+\frac{\sigma_{r}\left(t\right)-\sigma_{\theta }\left(t\right)}{r\left(t\right)}=0,$$

with σ representing the IWS in the corresponding directions. Thickness integration with the σr ri=p as a boundary condition on the luminal surface ($r=r_{i}$), gives

(5) $$p\left(t\right)=\int_{r_{i}\left(t\right)}^{r_{o}\left(t\right)}\left(\sigma_{\theta }\left(t\right)-\sigma_{r}\left(t\right)\right)\frac{dr}{r\left(t\right)}.$$

This equation can be rewritten in reference configuration by observing that r=λR and $dr={\left(\lambda \kappa \right)}^{-1}dR$ from Equations (2) and (3), with the constitutive relation [17,18] $\sigma_{\theta }-\sigma_{r}=\lambda \;\partial W/\partial \lambda$:

(6) $$p\left(t\right)=\int_{R_{i}\left(t\right)}^{R_{o}\left(t\right)}\frac{1}{\kappa \lambda }\;\frac{\partial }{\partial \lambda }\left[\underset{k}{\sum }W^{k}\left(t\right)+W^{a}\left(t\right)\right]\frac{dR}{R\left(t\right)}.$$

Neglecting through thickness variation of the integrand with the thin-wall assumption, i.e., $h\left(t\right)<<r_{i}\left(t\right)$, yields

(7) $$p\left(t\right)=\frac{1}{\kappa \lambda }\ln\left(\frac{R_{o}}{R_{i}}\right)\frac{\partial }{\partial \lambda }\left(W\left(t\right)+W^{a}\left(t\right)\right),$$

where $W\left(t\right)$ is the total strain energy per unit area at time t representing passive forces in the vasculature. On the other hand, $W^{a}\left(t\right)$ is the SM work density representing active forces acting on the arterial wall. 

Strain energy per unit area can be described by modeling the artery’s ability to adapt to changes of mechanical environment to maintain a homeostatic state via vascular G&R [16]. This process occurs through removal of old vascular constituents and incorporation of new constituents, which can be described in the following time evolution form:

(8) $$M^{k}\left(t\right)=M^{k}\left(0\right)Q^{k}\left(t\right)+\int_{0}^{t}m^{k}\left(\tau \right)q^{k}\left(t-\tau \right)d\tau ,$$

where $M^{k}\left(t\right)$ describes the current mass per unit area for the vascular constituent k. The first term on the right side of the equation is the natural turnover of the initial mass. $Q^{k}\left(t\right)$ is the current remaining fraction for constituent k produced at time 0. The second term describes the natural turnover of the newly produced constituents, with $q^{k}\left(t-\tau \right)$ being the remaining fraction of the constituent k produced at time τ and mass production rate $m^{k}\left(\tau \right)$. Here, it is assumed that $Q^{k}\left(t\right)$ and $q^{k}\left(t\right)$ follow an exponential decay, i.e., $\exp\left(-\nu^{k}t\right)$ with rate constant $\nu^{k}$ capturing turnover of constituent k, i.e., $1/80\left[\mathrm{days}\right]$ for collagen and SM [19]. Elastin is assumed to neither degrade nor grow [20], i.e., $Q^{e}\left(t\right)=1$ and $q^{e}\left(t\right)=0$. 

For a constrained mixture, based on the mass-average principle, the total strain energy per unit area at time t is

(9) $$W\left(t\right)=\underset{k}{\sum }\left(M^{k}\left(0\right)Q^{k}\left(t\right)W^{k}\left(t,0\right)+\int_{0}^{t}m^{k}\left(\tau \right)q^{k}\left(t-\tau \right)W^{k}\left(t,\tau \right)d\tau \right),$$

where $W^{k}\left(t,0\right)$ and $W^{k}\left(t,\tau \right)$ denote the strain energy density that constituent $k\;\in \left(c,m,e\right)$ produces at times 0 and τ, respectively. The vessel is assumed a constrained mixture of collagen, SM, and elastin, i.e., c,m,e.

Active stress in the circumferential direction $\sigma^{a}\left(t\right)$ can be defined from a constitutive relation assuming that vascular SM is oriented nearly circumferentially [21] with zero active stress in the radial and longitudinal directions.

(10) $$\sigma^{a}\left(t\right)=\lambda \frac{\partial W^{a}\left(t\right)}{\partial \lambda },\;\sigma_{z}^{a}\left(t\right)=\sigma_{r}^{a}\left(t\right)=0.$$

The active contribution of circumferential stress is modulated as a linear function of generated stress per unit relaxed SM area, i.e., $\sigma^{a}\left(t\right)=\lambda^{m}S$. Here, G&R takes place on a relatively long timescale where the SM response is more importantly characterized by the phenotypic dedifferentiation between a contractile and a synthetic state [9,22,23] as compared to short-term calcium-driven cyclic interactions. This phenotypic shift has been reported as increased non-muscle myosin expression with decreased SM myosin [9] in response to coarctation-induced mechanical stimuli, including those derived from increased systolic, mean, and pulse BP in regions proximal to the coarctation. Although these arterial alterations are in the direction of increased medial thickness through sustained remodeling, the active contractile force is somewhat impaired possibly due to a phenotypic shift from the contractile to synthetic state [9]. Moreover, arterial thickening is at the expense of increased structural stiffness leading to augmented forward and backward pressure waves that further increase pulse pressure and BP [24,25]. This creates a positive feedback loop that may become irreversible leading to refractory hypertension even after removal of the CoA. 

We assume herein that phenotypic dedifferentiation is a continuous process in which the SM cell gradually gains or loses its contractile response according to the function $S=S\;\left(C\right)$, where C≥0 is the phenotypic modulation stimulus. It represents the accumulation of stimuli descending from alterations in the stretch field. The generated active stress S is, therefore, associated with the slowly varying tone under normal physiological conditions [26]. Since not all infinitesimal areas of SM experience the same stretch, the expression for active stress is adapted to a situation with variable $\lambda^{m}$, so that the total active stress difference $\sigma^{a}\left(t\right)-\sigma_{r}^{a}\left(t\right)$ is computed as a sum of active stress difference contributions from remaining infinitesimal area fractions produced at different times. To derive an equation for active stress differences, the derivative of Equation (9) is taken with respect to λ for SM constituent only. Multiplying both sides by λ then gives

(11) $$M^{m}\left(t\right)\lambda \frac{\partial W^{m}}{\partial \lambda }=M^{m}\left(0\right)Q^{m}\left(t\right)\lambda \left(t,0\right)dW^{m}\left(t,0\right)+\int_{0}^{t}m^{m}\left(\tau \right)q^{m}\left(t-\tau \right)dW^{m}\left(t,\tau \right)d\tau ,$$

where the notation $df\left(x\right)=df/dx$. Substituting Equation (10) gives

(12) $$M^{m}\left(t\right)\lambda \frac{\partial W^{a}\left(t\right)}{\partial \lambda }=M^{m}\left(0\right)Q^{m}\left(t\right)\lambda^{m}\left(t,0\right)S\left(C\right)+\int_{0}^{t}m^{m}\left(\tau \right)q^{m}\left(t-\tau \right)\lambda^{m}\left(t,\tau \right)S\left(C\right)d\tau ,$$

where $\lambda^{m}\left(t,0\right)$ and $\lambda^{m}\left(t,\tau \right)$ are the stretch ratio for SM constituents generated at times 0 and τ, respectively, and can be described in terms of stretch developed during growth, i.e., $\lambda \left(t\right)/\lambda \left(\tau \right)$, and pre-stretch of SM ${\hat{G}}^{m}$ that develops at the time of production, which is assumed to be a constant equal to 1.3 according to Beak et al. [27,28]:

(13) $$\lambda^{m}\left(t,\tau \right)=\frac{\lambda \left(t\right)}{\lambda \left(\tau \right)}{\hat{G}}^{m}.$$

The phenotypic modulation of contractile activity is taken to be

(14) $$S\left(C\right)=S_{M}\left(1-\exp\left(-C^{2}\right)\right),$$

where $S_{M}$ is the maximum stress capacity of SM in its fully contractile state and assumed to be a constant equal to 100 kPa [29]. For a target lumen radius ${\hat{r}}_{i}$, the stretch is $\hat{\lambda }={\hat{r}}_{i}/R_{i}$ and we refer to $\hat{\lambda }$ as the target homeostatic stretch. From the Hagen–Poiseuille law, the $\frac{\lambda }{\hat{\lambda }}=\frac{r_{i}}{\;{\hat{r}}_{i}}={\left(\frac{\;\hat{\phi }}{\phi }\right)}^{1/3}$ is available to G&R through WSS signaling from endothelial cells. Hence, the time window average phenotypic modulation stimulus, C, of the vascular SM depends only on this known ratio $\lambda /\hat{\lambda }$, giving

(15) $$M^{m}\left(t\right)\lambda \frac{\partial W^{a}\left(t\right)}{\partial \lambda }=M^{m}\left(0\right)Q^{m}\left(t\right)\frac{\lambda }{\lambda \left(0\right)}{\hat{G}}^{m}S\left(C\left[\frac{\lambda }{\hat{\lambda }}\right]\right)+\int_{0}^{t}m^{m}\left(\tau \right)q^{m}\left(t-\tau \right)\frac{\lambda }{\lambda \left(\tau \right)}{\hat{G}}^{m}S\left(C\left[\frac{\lambda }{\hat{\lambda }}\right]\right)d\tau .$$

Dividing both sides by λ gives

(16) $$\frac{\partial W^{a}\left(t\right)}{\partial \lambda }=\frac{{\hat{G}}^{m}}{{\langle \lambda \rangle }^{m}}S\left(C\left[\frac{\lambda }{\hat{\lambda }}\right]\right),$$

where the effect of the growth history is captured by a SM area-weighted harmonic mean of the stretch:

(17) $${\langle \lambda \rangle }^{m}=M^{m}\left(t\right)\times {\left[\frac{M^{m}\left(0\right)}{\lambda \left(0\right)}Q^{m}\left(t\right)+\int_{0}^{t}\frac{m^{m}\left(\tau \right)}{\lambda \left(\tau \right)}q\left(t-\tau \right)d\tau \right]}^{-1}.$$

The SM work density can then be formally defined as

(18) $$W^{a}\left(\lambda \right)=\frac{{\hat{G}}^{m}}{{\langle \lambda \rangle }^{m}}\int_{\hat{\lambda }}^{\lambda }S\left(C\left[\frac{\lambda \prime }{\hat{\lambda }}\right]\right)d\lambda \prime ,$$

where the lower limit of the integration is arbitrary owing to the constant term of the primitive function. In a homeostatic state, taking the limit t→∞ for Equation (17) gives ${\langle \lambda \rangle }^{m}\equiv \hat{\lambda }$, so that the SM work density in the homeostatic state becomes

(19) $${\hat{W}}^{a}\left(\lambda \right)=\frac{{\hat{G}}^{m}}{\hat{\lambda }}\int_{\hat{\lambda }}^{\lambda }S\left(C\left[\frac{\lambda \prime }{\hat{\lambda }}\right]\right)d\lambda \prime .$$

The derivatives of the SM work density (i.e., ${\hat{W}}^{a}$), according to the constitutive relation, give the active force in pathological conditions, which can then be normalized to its homeostatic value obtained from a normal hemodynamics. Here, it was assumed that impaired active response is linearly correlated to the phenotypic shift of SM from the contractile to synthesis state. Hence, impairment was quantified in the G&R model through the ratio of CoA to homeostatic active stress, named the impairment index, and compared to corresponding values obtained empirically using wire myography, i.e., percentage impaired contractile or relaxation behavior of CoA rabbits relative to the control group. 

It is worth mentioning that, since the hemodynamic modulation stimulus ($C\left[\frac{\lambda^{\prime }}{\hat{\lambda }}\right]$) is an unknown function, it was represented using a truncated Taylor expansion around the homeostatic condition, $\frac{\lambda^{\prime }}{\hat{\lambda }}=1$.

(20) $$C\left[\frac{\lambda }{\hat{\lambda }}\right]=\hat{C}+C^{\prime }\left(\frac{\lambda^{\prime }}{\hat{\lambda }}-1\right),$$

where $\hat{C}$ and $C^{\prime }$ are nondimensional constants representing the phenotypic modulation stimulus of SM under homeostatic and pathological conditions, respectively. Under homeostasis, $\hat{S}$ = *S*(*C*[1]) = S($\hat{C})$, which, according to Equation (14), gives $\hat{C}=\surd \left(-\ln\left(1-\hat{S}/S_{M}\right)\right)=0.8326$ for our choice of parameters. Therefore, we assumed $\hat{C}$ as constant and tuned C’ in the G&R model to replicate phenotypic modulation and associated active vascular impairment obtained empirically using an impairment index defined in myography results as detailed below.

### 2.2. Strain Energy Density Function for Vessel Constituents

Currently, we define a general form of a strain energy density function to describe the passive action of vessel constituents. In general, if it is assumed only one family of collagen and SM is circumferentially oriented, the strain energy density function for both can be defined similar to elastin using a hybrid neo-Hookean and Fung-type material [30]:

(21) $$W^{k}\left(\lambda ,\kappa \right)=\frac{c_{1}^{k}}{2}\left(\lambda^{2}+{\left(\kappa \lambda \right)}^{-2}+\kappa^{2}-3\right)+\frac{c_{2}^{k}}{4c_{3}^{k}}\left\{\exp\left[c_{3}^{k}{\left(\lambda^{2}-1\right)}^{2}\right]-1\right\},$$

where $c_{i}^{k},\;i\in \left(1,2,3\right)$ are constitutive parameters characterizing material properties of constituent $k\in \left(c,m,e\right)$. Now, we have all strain energy and SM work densities in terms of λ, which is a function of current and generation time, i.e., $\lambda \left(t,\tau \right)$. Hence, the governing equation of G&R can be derived and discretized for the numerical simulation described in the next section.

### 2.3. Simulation of Stress Mediated G&R

For a pressurized thin-walled incompressible elastic tube with radius r and thickness h, Hoop stress can be derived by force equilibrium in the radial direction:

(22) $$\sigma_{\theta }=\frac{pr_{i}}{h},$$

where $h\ll r_{i}$ and denote wall thickness and internal radius, respectively.

For laminar flow of a Newtonian fluid with viscosity, η, and velocity, u, the Hagen–Poiseuille equation describes WSS as

(23) $$\phi_{w}=\frac{4\eta u}{\pi r_{i}^{3}}.$$

Considering that, for a thin-walled tube, area can be described as $A≅2\pi r_{i}h$, rewriting Hoop stress and Hagen–Poiseuille in terms of A and dividing by the hemostatic state denoted by hat sign ($\hat{\;}$), one can derive the current to homeostatic area ratio in terms of mechanical stresses acting on the wall:

(24) $$\frac{\hat{A}}{A}=\frac{\sigma_{\theta }}{{\hat{\sigma }}_{\theta }}\;{\left(\frac{\phi }{{\hat{\phi }}_{\;}}\right)}^{2/3}.$$

This equation demonstrates how two known local stimuli, namely, the WSS and IWS, mediate to reproduce the ratio $\hat{A}/A$ and, thus, the appropriate direction of growth: increased rate of production for $\hat{A}/A>1$ and decreased rate of production for $\hat{A}/A<1$. Taylor series expansion of Equation (24) around the homeostatic state $\hat{A}/A=1$ gives

(25) $$\frac{\hat{A}}{A}\approx 1+\frac{\Delta \sigma_{\theta }}{{\hat{\sigma }}_{\theta }}+\frac{2}{3}\frac{\Delta \phi }{{\hat{\phi }}_{\;}}.$$

Stress-mediated remodeling theory identifies $K_{\sigma }={\hat{\sigma }}_{\theta }^{-1}$ and $K_{\phi }=\frac{2}{3}{\hat{\phi }}^{-1}$, and the basal production rate analogous to the definition of mass production rate as a linear function of mechanical stimulus is as follows [31,32]:

(26) $$m^{k}\left(t\right)=m_{0}^{k}\left(1+K_{\sigma }^{k}\Delta \sigma_{\theta }^{k}+K_{\phi }^{k}\Delta \phi \right),$$

with $m_{0}^{k}$ being the basal mass production rate of constituent $k\in \left(c,m,e\right)$.

In summary, Equation (26) governs the evolution of the vessel constituents through Equation (1) where the mass per unit area for each vessel constituent is defined. WSS can be derived either from the Hagen–Poiseuille law or from computational fluid dynamics (CFD) using a reduced-order 1D model (ROM). The intramural stress is quantified from constitutive relations and strain energy, and SM work densities are defined according to the growth history in the previous section, 0≤τ≤t. Since the right-hand side of Equation (26) depends on the growth history, Equation (1) is the integral form of a delay differential equation (DDE) that is solved using an explicit numerical scheme described below.

The distribution of remaining mass per unit area is defined as

(27) $$M^{k}\left(t,\tau \right)=m^{k}\left(\tau \right)q^{k}\left(t-\tau \right),\;t\geq 0,\;0\leq \tau \leq t,$$

such that $M^{k}\left(t,\tau \right)d\tau$ represents the amount of constituent k that remains at time t, and produced within the time interval $\left[\tau ,\tau +d\tau \right)$. It follows that

(28) $$M^{k}\left(t+\Delta t,\tau \right)=\frac{q^{k}\left(t+\Delta t-\tau \right)}{q^{k}\left(t-\tau \right)},$$

for any constant time-step Δt, and that

(29) $$M^{k}\left(t,t\right)=m^{k}\left(t\right)q^{k}\left(0\right)\to \;M^{k}\left(t,t\right)=m^{k}\left(t\right).$$

Time is discretized using time-step Δt, with the following discretization points:

(30) $$t_{n}=n\Delta t,\;\;\;n=1,\;2,\ldots ,N.\;$$

Then, $M^{k}$ becomes discretized as

(31) $$M_{n,j}^{k}=M^{k}\left(n\Delta t,j\Delta t\right),\;\;\;\;\;\;\;j=0,\;\ldots ,n.$$

From Equation (28), we also have

(32) $$M_{n+1,j}^{k}=b_{n,j}^{k}M_{n,j}^{k}\;\;,\;\;\;b_{n,j}^{k}=\frac{q^{k}\left[\left(n-j+1\right)\Delta t\right]}{q^{k}\left[\left(n-j\right)\Delta t\right]}.$$

An explicit numerical scheme is used to integrate the evolution of the vessel constituents using Equation (1) with Equation (26). Slowly varying quantities, such as $m^{k}\left(t\right)$ and ${\langle \lambda \rangle }^{m}$, are treated explicitly, while λ, which changes instantaneously due to changes in the controlling parameters $\hat{\lambda }\left(t\right)$ and $p\left(t\right)$, is treated implicitly. Therefore, integrals are approximated using a trapezoidal rule throughout. For a compact notation, we define a summation symbol used for the trapezoidal rule:

(33) $$\overset{n*}{\underset{i=m}{\sum }}a_{i}=\left\{\begin{array}{cc} 0 & m\geq n\\ \frac{1}{2}a_{m}+\overset{n-1}{\underset{i=m+1}{\sum }}a_{i}+\frac{1}{2}a_{n} & otherwise \end{array}\right..$$

The equilibrium Equations (7) and (16) give

(34) ∑k∂∂λWk n+G^nm〈λ〉nmSCλn+1λ^n+1+lnRiRoκλn+1pn+1=0.

This equation is solved numerically for $\lambda_{n+1}$ with ${\hat{\lambda }}_{n+1}$ as an initial guess using the MATLAB numerical solver (MathWorks, Natick, MA, USA). If the solution is not unique, the closest solution to ${\hat{\lambda }}_{n+1}$ is chosen [26].

The stress measure $\sigma_{n+1}$ is computed using stress difference terms quantified through differentiating strain energy or SM work density with respect to λ and then multiplying by λ:

(35) σn+1=∑kλn+1∂∂λWk n+λn+1G^nm〈λ〉nmSCλn+1λ^n+1.

For each constituent $k\in \left(c,m,e\right)$, the newly produced materials are computed according to Equations (26) and (29):

(36) $$M_{n+1,n+1}^{k}=m_{0}^{k}\left(1+K_{\sigma }^{k}\left(\sigma_{n+1}-{\hat{\sigma }}_{n+1}\right)+K_{\phi }^{k}\left(\phi_{n+1}-{\hat{\phi }}_{n+1}\right)\right).$$

Moreover, the remaining materials $M_{n+1,j}^{k},\;j=0,\;1,\ldots ,n$ from production at earlier times is computed using Equation (32). The mass per unit area of each constituent is calculated from Equation (1) using the trapezoidal rule:

(37) $$M_{n+1}^{k}=M^{k}\left(0\right)Q^{k}\left(n\Delta t+\Delta t\right)+\Delta t\overset{n+1*}{\underset{j=0}{\sum }}M_{n+1,j}^{k}.$$

The weighted harmonic mean of the stretch is also expressed through Equation (17) and the trapezoidal rule:

(38) $${\langle \lambda \rangle }_{n+1}^{m}=M_{n+1}^{m}{\left[\frac{M^{m}\left(0\right)}{\lambda \left(0\right)}Q^{m}\left(\left[n+1\right]\Delta t\right)+\Delta t\overset{n+1*}{\underset{j=0}{\sum }}M_{n+1,j}^{k}\frac{1}{\lambda_{j}}\right]}^{-1}.$$

Using the trapezoidal rule and Equation (9) for one constituent gives

(39) ∂∂λMkWk n+1=M0kQkn+1ΔtG^kλ0dWkλλ0G^k+Δt∑j=0n+1*Mn+1,jk G^kλjdWkλλjG^k,

which is a function of λ to be used for implicit calculation of $\lambda_{n+2}$ in the subsequent time-step. With these operations, all variables are obtained for the timestep $\left(n+1\right)\Delta t$, and the new functions $\frac{\partial }{\partial \lambda }\left(M^{k}W^{k}\right)$ are defined for use in the following timestep. Here, ${\hat{G}}^{k}$ represents the pre-stretch for constituent k and assumed to be constant and equal to 1.08 and 1.4 for collagen and elastin, respectively [28].

### 2.4. G&R Model Fitting

Constitutive parameters of the derived G&R model were tuned to simulate the kinetics of growth and remodeling in response to CoA-induced mechanical stimuli. More specifically, results of the simulations for arterial thickening, stiffening, and dysfunction were compared to measurements of these precursors of hypertension quantified among nine CoA groups (details of empirical measurements are provided in the next section). Constitutive parameters were tuned to minimize the following weighted objective function:

(40) $$\chi^{2}=\sum_{k}\omega_{k}SEE_{k},$$

where $SEE_{k}$ is the sum of square error of the model vs. empirically measured hypertension precursors with $k\;\epsilon \;\left\{stiffness,\;thickness,\;dysfucntion\right\}$, and $w_{k}$ represents the weight constant for the *k*-th hypertension precursor. A genetic algorithm was used in MATLAB [33] to solve the nonlinear optimization problem by setting the population size of 1000 and function tolerance of 1 × 10^−7^. Collectively, the resulting fit represented a mathematical model tuned to predict thickness evolution (from longitudinal Doppler ultrasound images) and terminal stiffness and dysfunction (from invasive final material testing and myograph analysis). Table 1 lists the G&R parameters in the model with references and/or values, where applicable.

### 2.5. Empirical Quantification of Hypertension Precursors

Under pathological conditions, G&R can be described through SM synthesis and collagen turnover [16] that are both assumed to be stress-driven [31] and can, therefore, be correlated to the CoA-induced mechanical stimuli. Identifying associated G&R constitutive parameters, thus, requires determination of (1) mechanical stimuli, and (2) arterial dysfunction for an array of cases with different severity and durations of CoA. For this purpose, a rabbit model of CoA accommodating various severities and durations of the mechanical stimuli was implemented [8]. Hypertension precursors including arterial thickening, stiffening, and dysfunction were quantified using an array of noninvasive and invasive experimental protocols [8].

#### 2.5.1. Experimental Protocol

**Figure 1:**
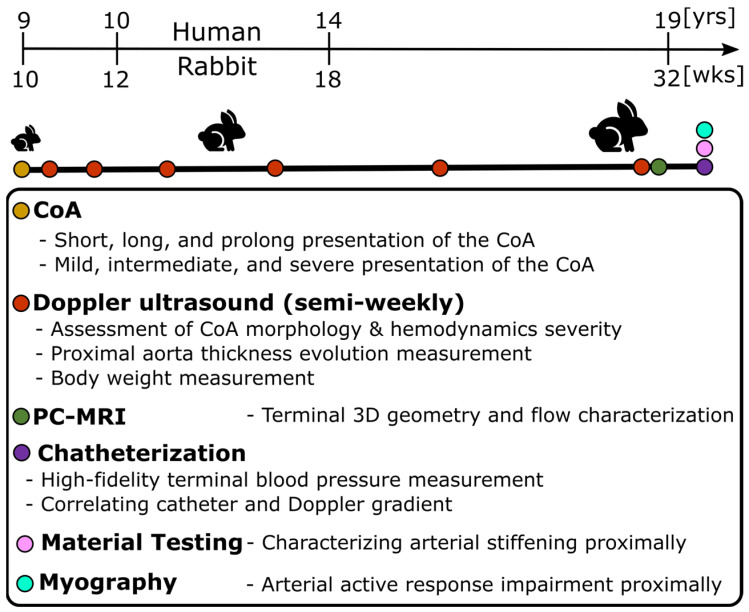
Experimental protocol to characterize hypertension precursors in response to CoA in rabbits. Male New Zealand White rabbits were randomly selected for a control group or designated to undergo descending thoracic CoA (nine study groups: three severities and three durations) via left thoracotomy in the third intercostal space. Weekly Doppler ultrasound imaging characterized local hemodynamic and morphology adaptation to CoA before PC-MRI prior to the final week. At the end of the protocol, invasive characterization was conducted for blood pressure measurement, and the aorta was dissected to quantify arterial stiffening and dysfunction through uniaxial extension testing and myograph analysis, respectively.

Hypertension precursors included (1) arterial thickening, (2) stiffening, and (3) dysfunction, which were quantified following previously published protocols for a rabbit model mimicking human CoA. Figure 1 summarizes the workflow for the experimental protocol and quantities measured using each modality. After IACUC approval, CoA was surgically induced under isoflurane anesthesia in New Zealand white rabbits (total 63; n = 5–11/group) by tying suture to varying diameters around the aorta at an age of ~10 weeks [8]. Tying the suture at different diameters, i.e., 2.06, 1.63, and 1.40 mm, resulted in various clinically important severities named mild (peak-to-peak CoA gradient ≤13 mmHg), intermediate (peak-to-peak CoA gradient 13–20 mmHg), and severe (peak-to-peak CoA gradient ≥20 mmHg). Importantly, the putative threshold suggestive of treatment for CoA in humans is ≥20 mmHg [37].

To investigate the effect of duration of the mechanical stimuli caused by the CoA, sutures with different dissolving properties, i.e., rapid dissolvable, dissolvable, and permanent, were used to present stenoses for different durations of ~2, 5, and 22 weeks named short, long, and prolonged CoA, respectively. Importantly, sutures were tied around the proximal descending thoracic aorta (distal to the left subclavian branch) where CoA most often presents. Together, this combination resulted in nine study groups (three severities and three durations) plus a control group.

#### 2.5.2. Temporal Monitoring of Morphology and Hemodynamics

**Figure 2:**
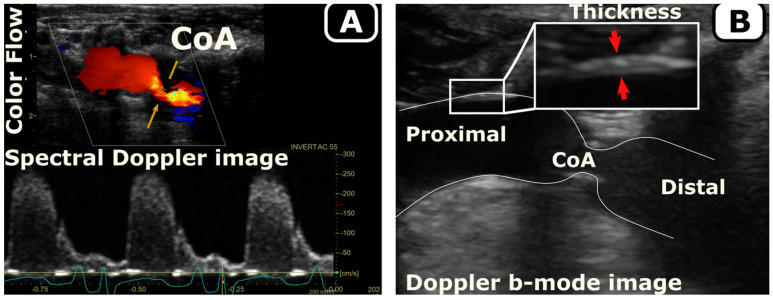
Quantification of hemodynamics (**A**) and morphology (**B**) in a rabbit model of CoA. Doppler ultrasound follow-up was performed weekly to quantify the temporal evolution of hemodynamic changes (**A**, spectral Doppler) and morphology (**B**, Doppler b-mode) in response to the CoA.

Using body weight at arrival and according to the growth rate for New Zealand White rabbits [38], age was estimated and then scaled to that of humans according to the duration of different phases of life [39] (Figure 1). Temporal hemodynamic and morphological evaluation was performed as a function of age using Doppler ultrasound imaging following protocols similar to human transthoracic echocardiography. Specifically, Doppler color flow and spectral Doppler images (Figure 2A) were used to identify pre- and post-stenotic flow and associated peak velocity for Doppler-based hemodynamic assessment, while b-mode imaging (Figure 2B) was used to quantify morphological properties, such as aorta diameter, wall thickness, and percentage area obstruction, using the protocols detailed elsewhere [8,9]. Moreover, phase-contrast magnetic resonance imaging (PC-MRI) was performed to characterize morphological properties, as well as quantify cardiac output and flow distribution to the main aortic branches [8,9]. Lastly, high-fidelity BP measurements were performed at the end of the protocol via invasive catheterization before harvesting of aortic tissue for further invasive characterization, e.g., material properties through uniaxial extension testing and vascular dysfunction through myograph analysis (Figure 1). Together, these empirical measurements were quantified as surrogates (i.e., precursors) of hypertension including arterial thickening, stiffening, and dysfunction, as detailed in the next subsections.

#### 2.5.3. Vascular Stiffening

To investigate coarctation-induced arterial stiffening, material properties were quantified in the proximal descending thoracic aorta (region between the left subclavian artery and the coarctation). This region was selected due to its exposure to adversely elevated coarctation-induced BP. Material properties were characterized through uniaxial extension testing (MTS Criterion Load Frame, MTS, Minneapolis, MN, USA) at 37 °C in an environmental chamber (MTS Bionix EnviroBath, Minneapolis, MN, USA). Tissues were dissected in the circumferential direction with a length-to-width ratio of ~2.6 and preconditioned by stretching to 10% of the gauge length. Extension testing was performed at 10 mm/min until hyperelastic behavior appeared. Results were interpreted in stress–strain curves to be compared to G&R model predictions quantified via Equation (34) at the experimental endpoint.

#### 2.5.4. Vascular Thickening

**Figure 3:**
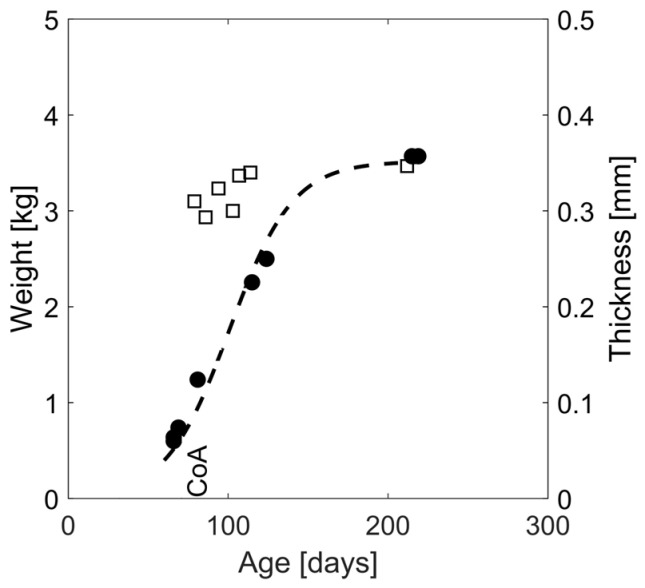
Representative thickness and body weight evolution over time for a CoA rabbit. Thickness (squares) was quantified as a function of age and through weekly ultrasound imaging at follow-up where 2D sagittal sections of the aorta were identified in b-mode images, and triplicate thickness measurements were performed with mean values shown at each timepoint. Body weight evolution was extrapolated using a sigmoid fit (dashed line) to the measured body weights (circles).

Doppler b-mode images were used to quantify aortic wall thickness in the proximal descending thoracic aorta (Figure 2B). All thickness measurements were made in triplicate with the mean value reported for each date. Body weight was also measured weekly using a scientific scale. Lastly, the thickness evolution was normalized to interpolated body weights at each timepoint assuming scaling of the body weight according to a sigmoid function (Figure 3). The sigmoid function was selected specifically to allow for a relatively low initial growth rate during the gestation period (~32 days) assuming an average of 50 g for newborn rabbit weight [39], as well as stable body weight, upon reaching adulthood.

#### 2.5.5. Vascular Dysfunction

Vascular specimens (3–4 mm rings) were carefully sectioned from the proximal descending thoracic aorta and assessed for vascular dysfunction using a protocol described previously [8]. Briefly, experimental assessment of active vascular dysfunction was conducted to observe SM contraction via phenylephrine (PE) in a half-log increasing dose responses from 10^−9^ to 10^−5^. To assess endothelium-dependent relaxation via nitric oxide (NO), arteries were precontracted with PE to the EC_50_ concentration. Once plateau was achieved, cumulative addition of the endothelium-dependent muscarinic receptor agonist acetylcholine (ACh; 10^−9^ to 10^−5^ M) was initiated, and relaxation response curves were quantified as a percentage of precontracted active tension. Area under the dose response curves (AUC) were then quantified as an aggregate measure of vasoactive dysfunction and normalized to that of the control group in a normalized quantity we refer to as the impairment index. All quantifications were performed in duplicate for paired channels with mean values reported for each rabbit. The impairment index was used as a quantitative nondimensional measure of active vascular impairment due to shift in SM phenotype to the dedifferentiated state and underlying endothelial dysfunction. This index was also quantified computationally using the CoA-to-control SM active stress ratio (Equation (18)) and used as a tool to tune the phenotypic modulation stimuli constant C′ in the G&R model.

#### 2.5.6. WSS and IWS Evolution

**Figure 4:**
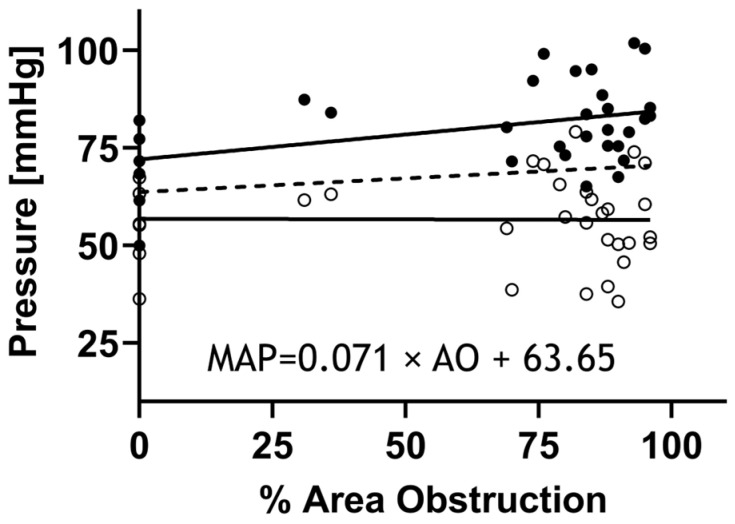
High-fidelity BP measurements proximal to CoA via catheterization. Rabbits (n = 55) were anesthetized for measurement of the BP above the coarctation prior to tissue harvest using high-fidelity pressure transducers (Harvard Apparatus, Holliston, MA, USA) attached to 0.86 mm ID noncompliant fluid-filled catheters inserted into the right common carotid artery and advanced to the aortic arch. Systolic blood pressure (SBP: black circles), mean arterial pressure (MAP), and diastolic blood pressure (DBP: open circles) were then quantified from the BP waveforms. Linear regression was then used to mathematically model MAP (dashed line) as a function of percent area obstruction (formula).

WSS (ϕ) and IWS (σ) are among the most important stress components deriving the rate of stress-mediated growth and remodeling as defined by Equation (26). WSS evolution was calculated on the basis of empirical data from the proximal descending thoracic aorta by implementing peak Doppler velocity readings in the Hagen–Poiseuille law assuming a parabolic velocity profile (Equation (23)). Additionally, IWS in the circumferential direction was quantified from the force equilibrium (Equation (22)). The radius and thickness in this equation were quantified from Doppler b-mode images as described earlier. As mentioned above, mean arterial pressure, i.e., MAP (p), was quantified through high-fidelity BP measurements using catheterization and described as a linear function of percentage area obstruction (Figure 4) determined from Doppler ultrasound images.

### 2.6. Statistical Analysis

Descriptive statistics are presented for continuous variables as the mean ± standard error of the mean (SEM). Unbalanced one-way analysis of variance (ANOVA) was used to assess significant differences across groups using Dunnett’s multiple comparison post hoc analysis with 5% level of significance in GraphPad Prism version 9.5.1, GraphPad Software, San Diego, CA, USA, www.graphpad.com (accessed on 12 October 2022). Pearson’s correlation and linear regression analysis examined the relationships and r-squared for goodness-of-fit. G&R model prediction was validated through impairment index values obtained from rabbits not used for fitting and in terms of percentage error and R-squared. D’Agostino and Pearson, Anderson–Darling, Shapiro–Wilk, and Kolmogorov–Smirnov tests were all used to assess the normality of prediction errors at the 0.05 significance level.

## 3. Results

**Figure 5:**
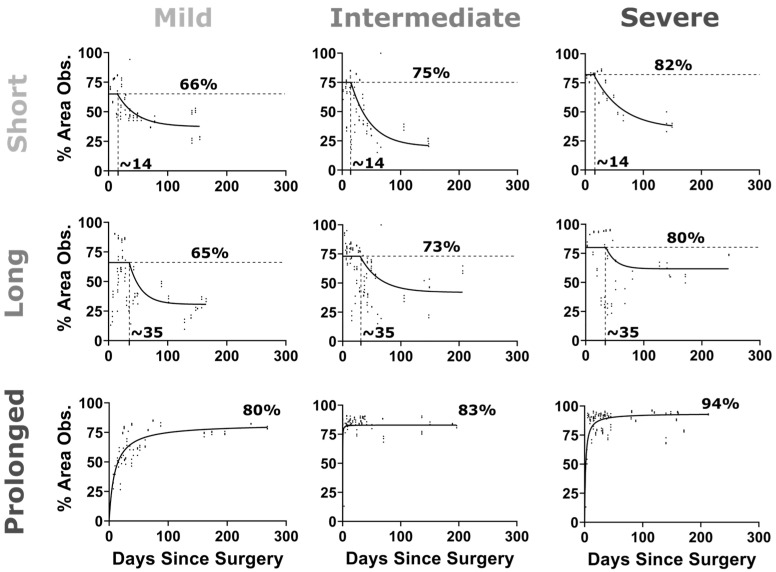
Percentage area obstruction vs. days after CoA surgery. CoA morphology was evaluated on a weekly basis noninvasively using ultrasound imaging. Proximal aorta and CoA diameters were quantified in triplicate, and the average value was plotted (scatter) at each date. The percentage area obstruction (%AO = 100 × (D_prox_^2^ − D_CoA_^2^)/D_prox_^2^) was, therefore, quantified and plotted vs. days after surgery. For the dissolving sutures (i.e., short and long duration CoA groups), a plateau followed by exponential decay function was fitted to the %AO, while, for the permanent suture (i.e., prolonged CoA group), an exponential function was used to fit the data.

Following the use of permanent suture in the prolonged CoA groups, the stenosis was present for ~22 weeks, resulting in adversely elevated BP. In contrast, degradation of the suture in the short and long CoA groups restored aortic diameter and, consequently, also restored associated elevation in BP. Area obstruction levels vary as a function of experimental group as shown in Figure 5 due to rabbit growth and resorption of dissolvable sutures.

**Figure 6:**
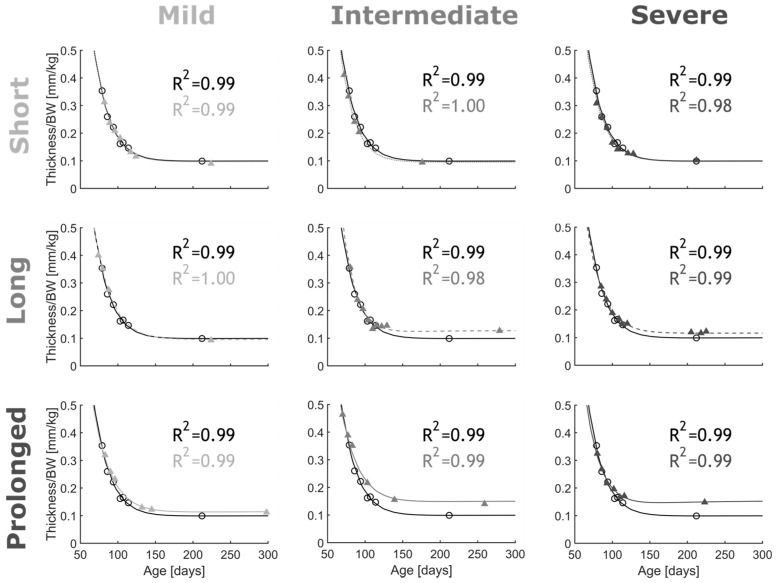
Thickness evolution for CoA rabbits (scatter) and G&R model predictions (lines). Coarctation-induced arterial thickening (shades of gray) was quantified from Doppler ultrasound b-mode images. To compare with control group, thicknesses were normalized to body weight (black). CoA groups included all combinations of severity (mild, intermediate, and severe) and duration (short, long, and prolonged) studied. G&R model constitutive parameters were tuned to fit empirical measurements (n = 2/group) with adjusted R^2^ characterizing goodness of fit.

*Arterial thickening.* Figure 6 shows the normalized thickness evolution for the CoA vs. control groups over the course of the protocol. Normalized thickness initially decreased in all rabbit groups and eventually plateaued with body weight after ~150 days of age. Overall, arterial thickening was observed in intermediate to severe CoA (peak-to-peak CoA gradient ≥13 mmHg) when presented for long (~5 weeks) or prolonged durations (~22 weeks). The pattern of arterial thickening was similar to the control group for short presentation of the CoA (i.e., all severities for ~2 weeks). In the mild CoA group (peak-to-peak CoA gradient <13 mmHg); however, thickness for the prolonged CoA group was slightly larger than that of the control at the plateau. After parameter calibration, the G&R model successfully replicated thickness evolutions. Table 2 reports the parameters of the G&R model obtained after the optimization procedure. The adjusted R-squared values for the resulting thickness evolution fits were in the range of 0.98 to 1.00 for all groups.

**Figure 7:**
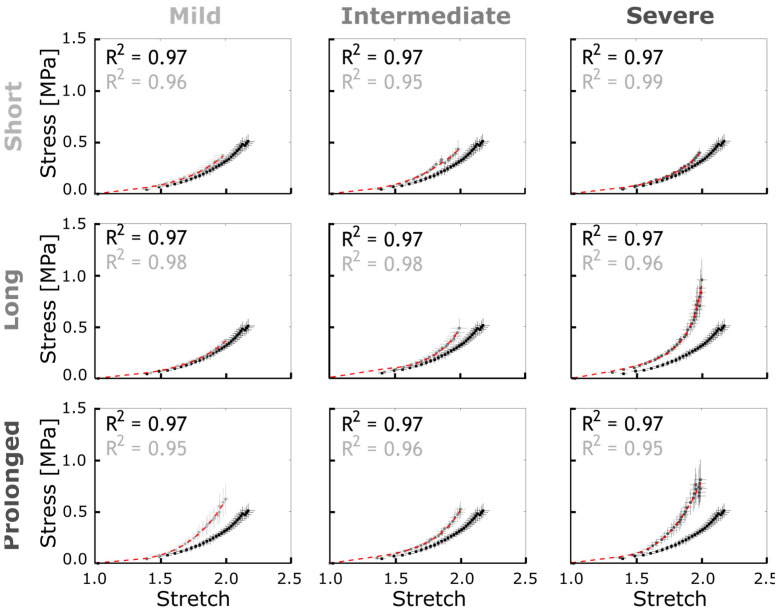
Empirical stress–stretch curves for CoA (gray) vs. control (black) groups and G&R model prediction (red). Error bars representing the SEM were quantified and averaged at each strain energy level discretized over the range of 0 to 3 MPa. The constitutive material parameters identified are listed in Table 2.

*Arterial stiffening.* Figure 7 represents the empirical stress–strain curves quantified in the proximal descending thoracic aorta exposed to adversely elevated BP in CoA groups. Results from each CoA group are compared to the control group in each plot. Overall, the pattern of arterial stiffening was observed by increased stress at the same stretch ratio for groups exposed to a prolonged CoA (~22 weeks). This was also observed in the long CoA groups (~5 weeks), but only for the intermediate and severe CoA (i.e., peak-to-peak CoA gradient ≥13 mmHg). To investigate the rate of stress-mediated remodeling and its effect on the vessel stiffness, constitutive parameters for collagen, SM, and elastin were tuned to fit the empirically measured stress–stretch curves at the end of the protocol (Figure 7, red lines). The constitutive material parameters obtained for c_1_, c_2_, and c_3_ are listed in Table 2.

**Figure 8:**
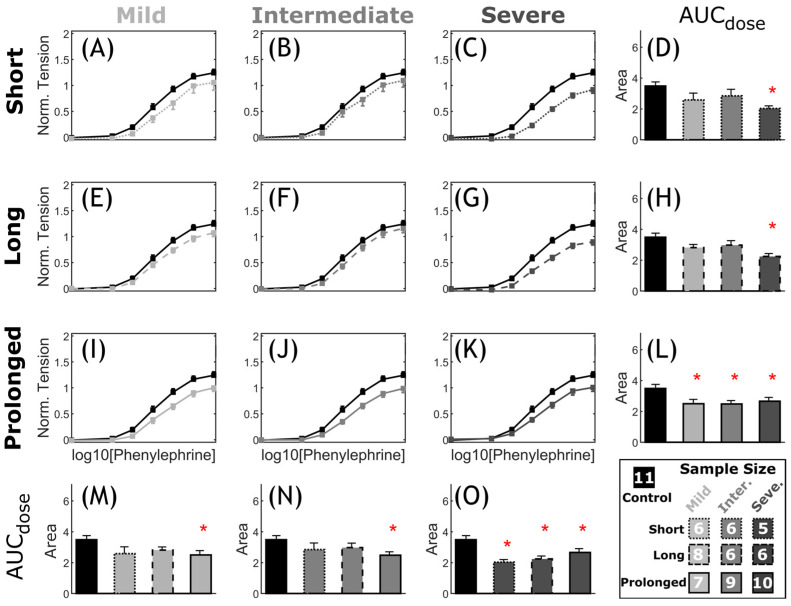
Comparative representation of active contraction response to PE in CoA vs. control. Dose response curves (myography) by increasing half-log dose of phenylephrine (PE: administered every 5 min). The measured contractile force was normalized to maximum K^+^ contraction achieved for each segment. The nine CoA groups consist of all combinations of severity (mild, intermediate, and severe: different gray levels) and duration (short, long, and prolonged: different line styles) studied, compared to the control group (black) in each plot (**A**–**C**,**E**–**G**,**I**–**K**). Bar plots (**D**,**H**,**L**–**O**) represent the area under each dose–response curve (AUC). Error bars represent the SEM. * Significant difference compared to the control group in a two-tailed Student’s *t*-test with a 5% significance level, assuming similar variation among the groups.

*Vascular dysfunction.* Overall, CoA rabbits showed impaired vascular response to half-log increasing dose of PE and ACh as compared to the control group. The extent of vascular impairment showed patterns correlating with both severity and duration of the CoA-induced mechanical stimuli. Figure 8 shows the normalized effective force response and associated AUC (Figure 8D,H,L–O) for CoA vs. control groups. The normalized effective force showed an intact logistic contraction response in the control rabbits peaking at 1.24 ± 0.07, whereas CoA groups peaked to smaller normalized tension values ranging from 0.90 ± 0.06 to 1.09 ± 0.13. This diminished contractile capacity was also observed through AUC among CoA rabbits (Figure 8D,H,L–O). For example, when CoA was at the most severe level, i.e., peak-to-peak CoA gradient ≥20 mmHg, the AUC was significantly smaller than in control rabbits, regardless of duration (Figure 8L). Similarly, when CoA was present for the prolonged period (~22 weeks), significant impairment was observed in AUC, regardless of severity (Figure 8O). A nonsignificant trend toward impaired PE contraction was observed in all other groups.

**Figure 9:**
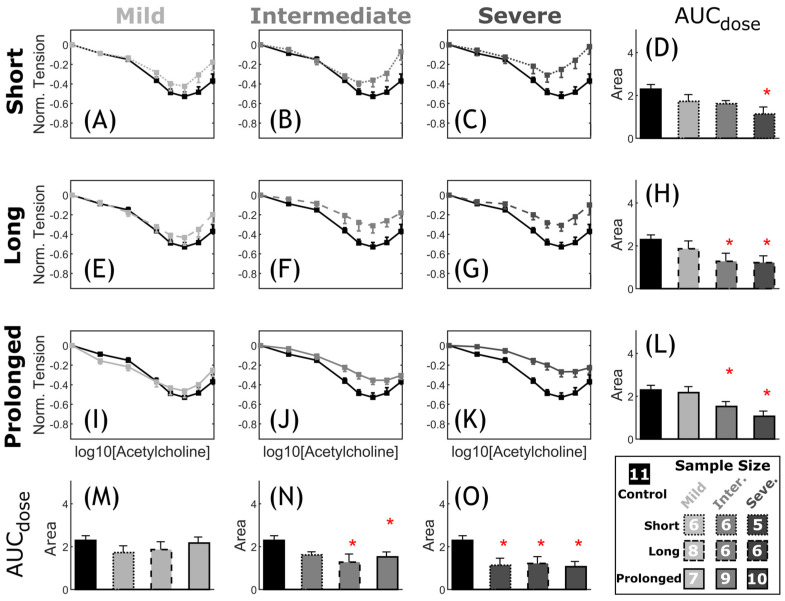
Comparative representation of CoA vs. control active relaxation response to ACh. Dose–response curves (myography) by increasing the half-log dose of acetylcholine (ACh: administered every 5 min). Relaxation was measured relative to the initial contraction (EC_50_ concentration). The nine CoA groups consisted of all combinations of severity (mild, intermediate, and severe: different gray levels) and duration (short, long, and prolonged: different line styles) studied and compared to the control group (black) in each plot (**A**–**C**,**E**–**G**,**I**–**K**). Bar plots (**D**,**H**,**L**–**O**) represent area under each dose–response curve (AUC). Error bars represent SEM. * Significant difference compared to the control group in a two-tailed Student’s *t*-test with 5% significance level assuming similar variation among the groups.

Figure 9 shows arterial relaxation curves in response to the endothelium-dependent agonist ACh for the descending thoracic aorta proximal to the CoA (exposed to adversely elevated coarctation-induced BP). Overall, control rabbits (black, n = 11) showed intact endothelial function with relaxation to 53% ± 4% of the precontraction force at 10^−7^ M ACh concentration. However, this vasodilatory action of ACh was impaired in CoA rabbits (gray, n = 5–10). The extent of impaired ACh relaxation correlated with the severity of CoA-induced mechanical stimuli. More specifically, significant decreases in AUC (Figure 9D,H,L–O) were observed in the severe CoA group (peak-to-peak CoA gradient ≥20 mmHg) regardless of the duration of the CoA (Figure 9L). Consequently, the quantified impairment index (CoA-to-control AUC ratio) was 0.52 ± 0.27, 0.55 ± 0.13, and 0.52 ± 0.16 for short, long, and prolonged duration groups, respectively. Rabbits with intermediate CoAs also developed impaired ACh relaxation response that was significant when exposed for >2 weeks, i.e., long and prolonged duration groups (Figure 9N). Associated impairment indices were 0.67 ± 0.19 and 0.85 ± 0.08, respectively. A trend toward impairment was observed in the mild CoA groups, but differences did not reach significance (Figure 9M).

**Figure 10:**
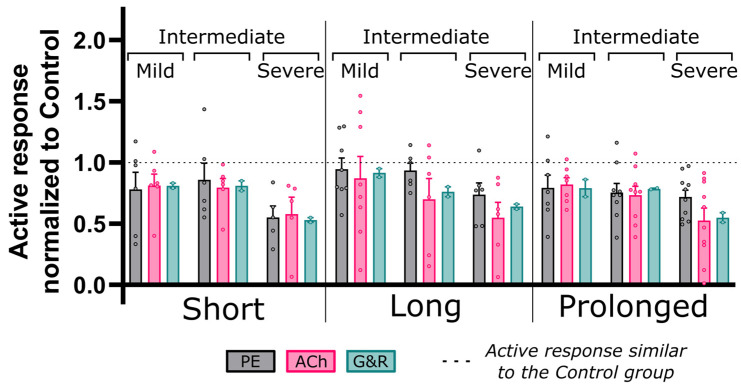
Coarctation-induced active vascular response normalized to the control group. Area under the active dose–response curves (AUC) for each CoA group normalized to that of the control. Dose–response curves were quantified via wire myography and a half-log increase in phenylephrine (PE, gray) and acetylcholine (Ach, pink) to characterize NO active response in SM contraction and relaxation, respectively. A similar index was also quantified by taking the ratio of active stress per unit relaxed SM for CoA relative to control groups using the G&R framework (green) for two rabbits per group. G&R constitutive parameters were tuned to achieve minimum error to empirically quantified active response, i.e., PE and Ach. No significant differences were observed between results from empirical and G&R through two-tailed Student’s *t*-test with a 5% significance level.

Phenotypic modulation was characterized computationally through its stimulus term ($C\left[\lambda^{\prime }/\hat{\lambda }\;\right]$) for SM as a function of the current to homeostasis (i.e., control) stretch ratio ($\left[\lambda^{\prime }/\hat{\lambda }\;\right]$). As indicated in Equation (20), $\hat{C}$ and $C^{\prime }$ are the two nondimensional constants that determine the phenotypic modulation in SM proliferation and impaired active response. Figure 10 shows active stress per unit relaxed SM normalized to that of the control group (impairment index) and compared to the corresponding empirical measurements of PE and ACh myograph results. Overall, PE contraction and ACh relaxation were both impaired with indices ranging from 0.55 to 0.94 (PE), and from 0.52 to 0.93 (Ach), as shown in Figure 10. Interestingly, this index correlated with the severity and duration of the mechanical stimuli caused by the CoA with values as low as 0.52 observed in the most severe and prolonged CoA groups. Phenotypic modulation stimulus constitutive parameters ($C^{\prime }$) tuned in the G&R model (n = 2) to replicate impairment quantified from wire myography are summarized for each group in Table 3.

## 4. Discussion

A G&R framework was implemented on the basis of previously developed models [15,16,19,26,40,41] to describe the evolution of arterial wall constituents in response to coarctation-induced mechanical stimuli. Parameters were fit to empirically quantified hypertension precursors among CoA rabbits. Underlying assumptions included thin-walled tube theory, constrained mixture theory, finite elasticity, Hagen–Poiseuille’s law assumptions, and long timescale phenotypic shift of SM from the contractile to synthetic state. Empirical measurements used to tune the model parameters included Doppler ultrasound imaging, uniaxial extension testing, high-fidelity invasive BP measurement, and wire myograph that together quantified the time evolution of arterial thickening, stiffening, and vasoactive dysfunction in response to mechanically induced CoA within a range of severities and durations seen clinically.

In general, good agreement was observed between G&R model prediction and empirical hypertension precursors among nine CoA study groups. Specifically, adjusted R-squared values >0.98 were obtained for thickness evolution (Figure 6), as well as >0.95 for arterial stiffening (Figure 7). There was also a good agreement between G&R predictions for an SM impairment index and empirical SM contraction and relaxation impairment indices obtained from wire myography (Figure 10).

Pathophysiological indices of arterial remodeling such as stiffening, thickening, and vasoactive dysfunction are strong predictors of adverse cardiovascular events and target organ damage of the heart, kidney, and brain [42]. Excessive increases in BP and pulsatility are believed to be the main hemodynamic factors triggering a cycle of events due to damaging effects on vascular function, and contributing to pathological arterial remodeling. Adversely elevated BP and pulsatility cause elevated IWS, causing increased stretch acting on collagen and the extracellular matrix. Additionally, increased cyclic stretch was shown and confirmed in the current study to stimulate a phenotypic shift in SM from the contractile to synthetic state. Moreover, these pathological conditions also affect endothelial function through adversely altered WSS [36,43,44]. Hence, they can be used as surrogates to modify currently debated pressure-based intervention guidelines toward preventing refractory hypertension.

**Figure 11:**
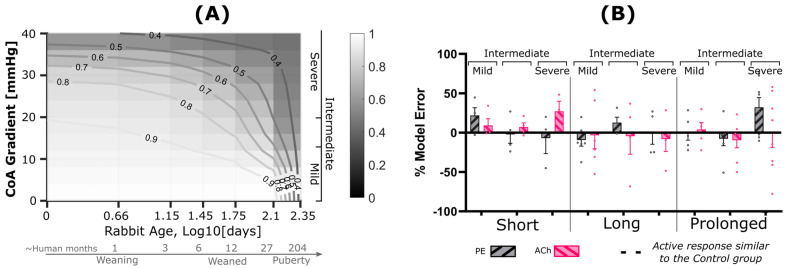
G&R model prediction (**A**) and prediction error (**B**) relative to the measured impaired vascular response. (**A**) Contour plot showing impairment index quantified by taking the ratio of active stress per unit relaxed SM for CoA relative to control using the G&R framework. Contour lines indicate regions of constant impairment indices. CoA severity, shown on the vertical axis, is characterized through peak-to-peak trans-coarctation BP gradient (BPG), whereas duration is represented on a logarithmic scale of age in days. (**B**) Model error relative to measured impairment index, i.e., area under the active dose–response curves (AUC) for each CoA group normalized to that of the control. Dose–response curves were quantified via wire myography and half-log increase of phenylephrine (PE, gray) and acetylcholine (Ach, pink) to characterize NO active response in SM contraction and relaxation, respectively. Model error was then quantified as a percentage relative to the empirical PE and Ach impairment indices. Rabbits used in the tuning process were excluded for model prediction error.

On one hand, identifying the kinetics of pathophysiological responses is challenging in CoA patients mainly due to antihypertensive agents administered, patient heterogeneities making it difficult to differentiate the severity of hemodynamic perturbations experienced, and time once irreversible vascular remodeling occurred. On the other hand, animal models allow for mechanistic investigation of vascular stiffening, thickening, and dysfunction under experimentally controlled conditions to characterize the kinetics of growth and remodeling. Importantly, the tuned model presented here can now be used as a computational tool to identify the severity and duration of the mechanical stimuli leading to hypertension precursors at different phases of rabbit life analogous to that of human. Particularly at younger ages that are difficult to study experimentally, the newborn and weaning phases can be computationally investigated using the G&R model presented. For example, Figure 11A shows the impairment index in the clinically important range of severity, as well as critical age phases. The lines of constant impairment index shown can be used as a map for CoA assessment considering both severity of the narrowing and rabbit age. Particularly, an active response <50%, i.e., impairment index <0.50, indicates nonviable tissue as reported by Lopez et al. [45]. Using this criterion, our results indicate that a peak-to-peak CoA gradient of >35 mmHg, even when exposed shortly, can permanently diminish the active arterial response (Figure 11A). The current model predictions show that such functional impairment is expected for a variety of CoA severity and durations including a short exposure to >30 mmHg peak-to-peak CoA gradient, as well as prolonged exposure to a milder CoA gradient (~10 mmHg). Interestingly, in ages younger than the rabbits studied, i.e., log_10_(days) < 1.75, i.e., ~70 rabbit days of age (or ~1 human year), model extrapolation showed arterial impairment being strongly correlated to severity of the CoA gradient, thus emphasizing the importance of early intervention to prevent impaired vascular function. This finding further emphasizes the application of such predictive models in clinical decision making, where both severity and duration of the coarctation-induced mechanical stimuli contribute to hypertension precursors developing in the aorta and major branches.

Figure 11B shows active response prediction errors for impairment indices representing NO active response in SM contraction and relaxation. Variability in prediction error was observed which was mainly considered to be inherited from biological variability of the impairment index reported in Figure 10. Statistical assessment of errors showed a normal distribution of across all study groups for both contraction and relaxation impairment indices using four normality tests mentioned in the methods section with *p*-values > 0.1.

The current results should be interpreted relative to several potential limitations. In particular, high-fidelity hemodynamic simulation is challenging to perform longitudinally as 3D reconstruction of the aorta over the course of the disease requires multiple MRI acquisitions and often computationally heavy [46,47]. We, therefore, quantified WSS and IWS from the Hagen–Poiseuille law and force equilibrium using empirical measurements of local hemodynamics over the course of disease development. Additionally, the large number of unknowns in the formulation derived for G&R requires a large sample size, and that is challenging to achieve due to the time and cost of experimental procedures needed for longitudinal monitoring of hypertension precursors using our preclinical model. Therefore, many of the constitutive parameters were assumed on the basis of the literature followed by tuning of the stress-mediating G&R parameters (Table 3) to replicate empirical results. It is also worth mentioning that the pathophysiological response of the aorta is also affected by concomitant morphological anomalies including the bicuspid aortic valve, aortic arch/isthmus hypoplasia, and long-segment coarctation. The associated effect on local hemodynamic and aortic remodeling remains to be investigated in future work.

## 5. Conclusions

Results showed that the implemented G&R model could accurately represent hypertension precursors among the studied groups of CoA rabbits. Therefore, the G&R model allows for computational identification of the adverse vascular response as a function of the severity and duration of coarctation-induced stimuli. Clinical translation of this predictive computational model can help in modifying current intervention thresholds and preventing irreversible hypertension precursors that seem to persist after CoA treatment using current clinical guidance.

## Figures and Tables

**Table 1:** Constitutive parameters in the derived G&R framework.

Parameter	Description	Reference	Value
$\nu^{c}$	Collagen turnover rate	Rhobin et al. [21]	1/80 [1/days]
$\nu^{s}$	SM turnover rate	Rhobin et al. [21]	1/80 [1/days]
$Q^{e}$	Initial elastin decay kinetics	Halayko et al. [22]	1
$q^{e}$	Remodeled elastin decay kinetics	Halayko et al. [22]	0
${\hat{G}}^{m}$	SM pre-stretch	Holzapfel et al. [34]	1.3
$S_{M}$	Maximum stress capacity of SM in the fully contractile state	Conn et al. [33]	100 [kPa]
$\hat{C}$	Phenotypic modulation stimulus parameter at normotensive condition	Lindstrom et al. [26]	0.8326
C′	Phenotypic modulation stimulus parameter at hypertensive condition	Touyz et al. [35]	[0.1, 100]
$c_{1}^{k}$	$\mathrm{Neo-Hookean}\;\mathrm{material}\;\mathrm{parameter}\;\mathrm{for}\;\mathrm{constituent}\;k\in \left\{c,s,e\right\}$	Wu et al. [20]	
$c_{2}^{k}$	$\mathrm{Exponential}\;\mathrm{material}\;\mathrm{parameter}\;\mathrm{for}\;\mathrm{constituent}\;k\in \left\{c,s,e\right\}$	Wu et al. [20]	
$c_{3}^{k}$	$\mathrm{Exponential}\;\mathrm{material}\;\mathrm{parameter}\;\mathrm{for}\;\mathrm{constituent}\;k\in \left\{c,s,e\right\}$	Wu et al. [20]	
${\hat{G}}^{c}$	Pre-stretch for collagen	Rachev et al. [32]	1.08
${\hat{G}}^{e}$	Pre-stretch for elastin	Rachev et al. [32]	1.4
$K_{\sigma }^{c}$	G&R collagen kinetic parameter for hoop stress	DePaola et al. [36]	$[2.0,\;18]\times 10^{-6}$
$K_{\phi }^{c}$	G&R collagen kinetic parameter for WSS	DePaola et al. [36]	$[0.8,\;8]\times 10^{-6}$
$K_{\sigma }^{s}$	G&R SM kinetic parameter for hoop stress	DePaola et al. [36]	$[2.0,\;18]\times 10^{-6}$
$K_{\phi }^{s}$	G&R SM kinetic parameter for WSS	DePaola et al. [36]	$[0.8,\;8]\times 10^{-6}$
$m_{0}^{c}$	Basal net production rate of collagen	Halayko et al. [22]	[0, 10]
$m_{0}^{s}$	Basal net production rate of SM	Halayko et al. [22]	[0, 10]
$m_{0}^{e}$	Basal net production rate of elastin	Halayko et al. [22]	0

**Table 2:** G&R constitutive material parameters for collagen, SM, and elastin obtained from CoA rabbits (n = 2 per group).

Rabbit Group		Constitutive Parameters								
Duration (Weeks)	Severity (Peak-to-Peak CoA Gradient)	Collagen *			Smooth Muscle *			Elastin **		
		c_1_ ***	c_2_	c_3_	c_1_	c_2_	c_3_	c_1_	c_2_	c_3_
Short (~2)	Mild (<13 ^†^)	0.0	597 ± 91	11.7 ± 4.8	0.0	11.5 ± 3.3	2.3 ± 1.4	56.2 ± 21	0.0	NA
	Intermediate (13–20)	0.0	646 ± 101	6.40 ± 2.1	0.0	13.2 ± 4.3	3.7 ± 1.8	67.4 ± 18	0.0	NA
	Severe (≥20)	0.0	613 ± 94	7.70 ± 2.0	0.0	13.0 ± 3.9	1.9 ± 1.0	58.7 ± 23	0.0	NA
Long (~5)	Mild (<13)	0.0	629 ± 97	10.5 ± 3.8	0.0	10.1 ± 4.2	3.7 ± 1.9	59.3 ± 8	0.0	NA
	Intermediate (13–20)	0.0	673 ± 113	9.70 ± 4.0	0.0	14.2 ± 4.8	4.8 ± 2.1	63.8 ± 16	0.0	NA
	Severe (≥20)	0.0	716 ± 117	14.9 ± 5.0	0.0	18.9 ± 4.3	7.2 ± 2.8	72.9 ± 31	0.0	NA
Prolonged (~22)	Mild (<13)	0.0	692 ± 88	12.7 ± 3.6	0.0	13.8 ± 5.1	11.7 ± 4.8	65.4 ± 21	0.0	NA
	Intermediate (13–20)	0.0	708 ± 122	13.2 ± 4.5	0.0	15.1 ± 3.8	11.7 ± 4.8	61.4 ± 17	0.0	NA
	Severe (≥20)	0.0	721 ± 42	14.0 ± 5.2	0.0	15.2 ± 4.2	11.7 ± 4.8	68.1 ± 24	0.0	NA

* Collagen and smooth muscle were described by an exponential strain energy density function [30] that requires c_1_ = 0. ** Elastin was described by a neo-Hookean [17] strain energy density function that requires c_2_ = 0 and also drops c_3_ from Equation (21). *** c_1_ and c_2_ are in kPa, and c_3_ is nondimensional. Values represent the average ± SD. ^†^ Pressures are in units of mmHg.

**Table 3:** G&R constitutive parameters determining the kinetics of stress-mediated response to CoA.

Rabbit Group		Constitutive Parameters				
Duration (Weeks)	Severity (Peak-to-Peak CoA Gradient)	Collagen		Smooth Muscle		
		$K_{\sigma }$ (×10^−6^)	$K_{\phi }$ (×10^−6^)	$K_{\sigma }$ (×10^−6^)	$K_{\phi }$ (×10^−6^)	*C*′
Short (~2)	Mild (<13 ^†^)	3.10 ± 2.0 *	1.80 ± 1.2	5.30 ± 3.0	3.90 ± 1.5	10.1 ± 3.0
	Intermediate (13–20)	5.20 ± 1.1	2.90 ± 1.2	10.2 ± 4.5	4.10 ± 2.0	8.80 ± 2.1
	Severe (≥20)	3.70 ± 0.9	1.70 ± 0.5	7.10 ± 2.7	4.50 ± 1.8	10.0 ± 2.8
Long (~5)	Mild (<13)	4.30 ± 1.6	2.30 ± 1.2	5.60 ± 3.1	5.60 ± 2.7	9.60 ± 2.5
	Intermediate (13–20)	4.10 ± 2.1	2.00 ± 0.8	7.60 ± 2.7	4.60 ± 1.1	11.6 ± 1.7
	Severe (≥20)	5.00 ± 1.7	3.50 ± 1.3	9.60 ± 1.5	3.60 ± 1.6	8.60 ± 2.1
Prolonged (~22)	Mild (<13)	5.40 ± 2.5	2.80 ± 1.2	5.60 ± 2.2	3.60 ± 1.1	7.60 ± 2.7
	Intermediate (13–20)	4.60 ± 1.3	3.10 ± 1.9	12.60 ± 2.8	5.60 ± 2.9	9.60 ± 3.0
	Severe (≥20)	6.30 ± 2.2	3.40 ± 1.7	10.60 ± 3.0	4.60 ± 2.7	10.6 ± 1.9

^†^ Pressures are represented in mmHg. * Values represent average ± SD.

## Data Availability

Data is contained within the article.

## Abbreviations

AA	Ascending aorta
BP	Blood pressure
CFPG	Continuous-flow pressure gradient
CoA	Coarctation of the aorta
IWS	Intramural wall stress
MBE	Modified Bernoulli equation
PC-MRI	Phase-contrast magnetic resonance image
ROM	Reduced-order model
SBE	Simplified Bernoulli equation
SBE-RP	SBE minus recovered pressure term
WSS	Wall shear stress
